# Case report: Use of laparoscopic electrocoagulation rods in endovenous thermal ablation of saphenous trunks in the treatment of varicose veins of the lower extremities

**DOI:** 10.3389/fsurg.2023.1189568

**Published:** 2023-05-25

**Authors:** Ling Wang, Ya Wang, Xiaoyang Niu, Zhengzuo Lv, Bing Wang

**Affiliations:** ^1^ Department of Vascular Surgery, The Fifth Affiliated Hospital of ZhengZhou University, Zhengzhou University, Zhengzhou, China; ^2^ Department of Vascular Surgery, Nanyang Central Hospital, Nanyang, China

**Keywords:** varicose veins, endovascular procedures, electrocoagulation, ablation, application of new methods

## Abstract

Varicose veins of the lower extremities are a very common condition in vascular surgery. With advances in technology and medicine, minimally invasive endovenous thermal ablation has become the primary approach used to treat patients with moderate or severe varicose veins. Electrocoagulation for thermal ablation is a relatively simple and economical procedure, but standards vary according to location and some limitations exist. We report a case of a 58-year-old female patient with small saphenous varicose veins in the right lower extremity in which an electrocoagulation rod commonly used in laparoscopic surgery was innovatively used instead of a standard variable electrocoagulation device. The venous clinical severity score was used to assess changes in clinical symptoms before and 3 months after the procedure. The procedure was shown to have eliminated venous reflux, improved the patient's clinical symptoms, and venous function. This procedure may be a reliable option for future endovenous electrocoagulation thermal ablation procedures for varicose veins that are simple and convenient to perform.

## Introduction

Varicose veins of the lower extremities are a common condition in vascular surgery. Successful surgical treatment of patients with moderate or severe varicose veins with swelling and ulceration of the lower extremities involves eliminating all sources of venous return. Historically, standard treatment involved high ligation and stripping of the great saphenous vein (GSV). However, many surgeons have used endovascular techniques in recent years and achieved good results (1). Consequently, conventional open surgery with high ligation and stripping of the GSV is rarely performed by vascular surgeons today (2). Various guidelines recommend thermal ablation as the first treatment option for varicose veins in the lower extremities, since radiofrequency ablation (RFA) and endovenous laser ablation (EVLA) offers better outcomes and prognosis over conventional open surgery (3). Thermal ablation techniques, however, require specialised ablation equipment, which is more expensive and may limit its widespread use. Various studies have demonstrated the possibility of applying electrical energy to treat varicose veins in the lower extremities. With the recent paradigm shift and improved understanding of endovascular treatment, electrocoagulation (EC) may be an alternative to endovascular treatment and may present advantages, since EC has similar therapeutic efficacy to radiofrequency and laser ablation (4, 5). EC is compatible with current peripheral endovascular catheterisation devices and techniques, and its lower cost could allow more patients to access thermal ablation to treat endovascular varicose veins (6). In EC procedures reported in the literature, EC of the saphenous vein trunk is commonly performed using customised electrocoagulation catheters of various types in conjunction with a high-frequency electric knife (7, 8). In the present case,we report a 58-year-old woman, who had varicose veins in the right lower extremity for 9 years. After obtaining written informed consent from the patient, we innovatively used a laparoscopic EC rod, instead of a standard customised EC catheter, as an electrode for endovenous thermal ablation. Laparoscopic EC rods are economical, readily available, and are commonly used for tissue coagulation and wound haemostasis in laparoscopic surgery.

## Case description

**Figure 1:**
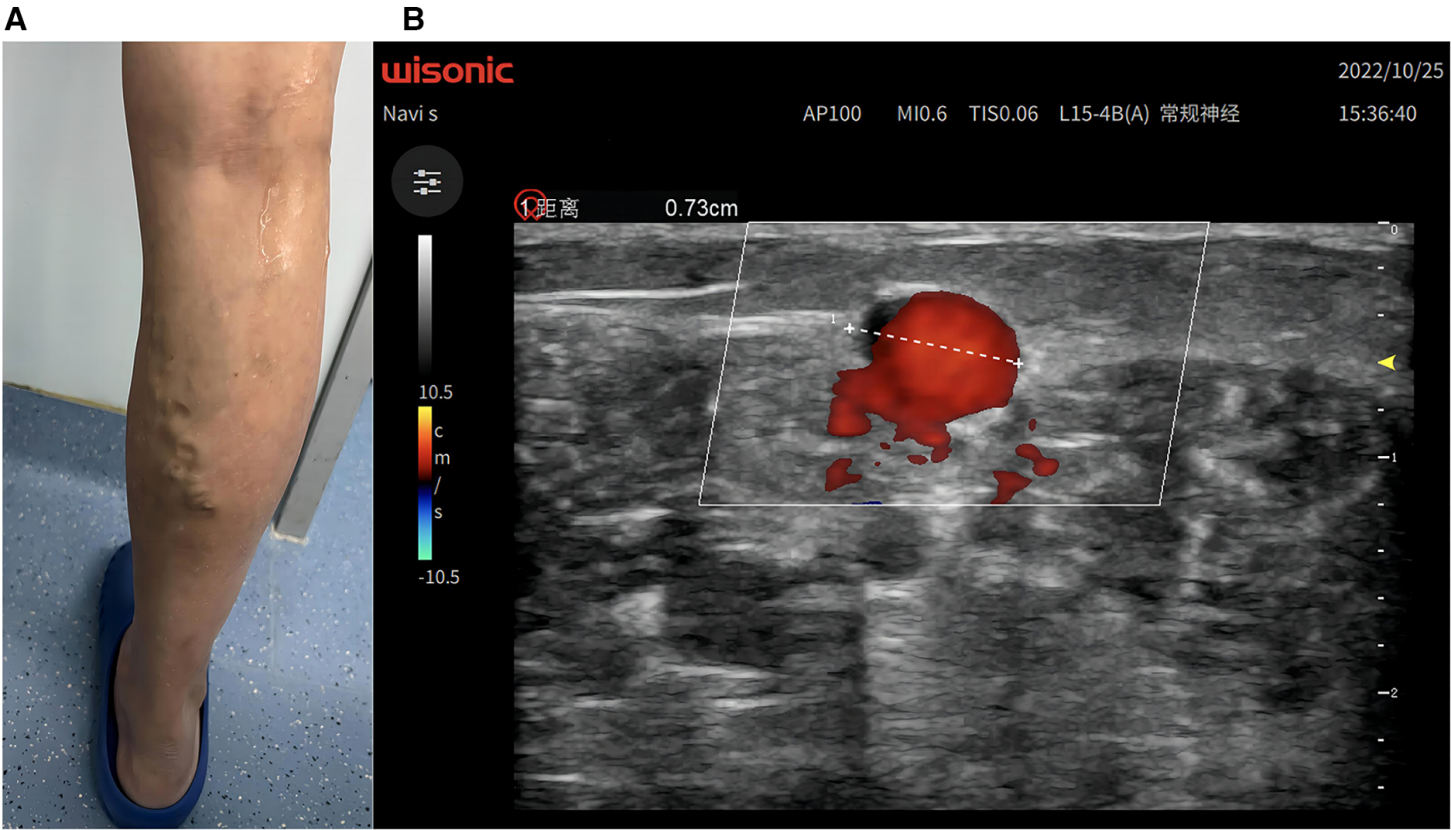
(**A**) Appearance before operation. (**B**) Preoperative ultrasound showed small saphenous vein regurgitation.

A 58-year-old woman, who had varicose veins in the right lower extremity for 9 years (Figure 1A), was admitted to the hospital with a venous clinical severity score (VCSS) score of 2. On physical examination, the superficial varicose veins in the medial posterior aspect of the right lower calf were negative on the Perthes test (deep femoral vein patency) and the Pratt test (venous valve function test). Colour ultrasound Doppler of vessels in the right lower extremity indicated severely inadequate closure of the right small saphenous vein valve and dilatation of the calf muscle veins (Figure 1B). According to the latest treatment guidelines (3), the patient was indicated for endovenous electrothermal ablation and sclerotherapy of the right lower extremity varicose veins. Following preoperative discussion and approval by the Institutional Review Board of the hospital, a unanimous decision was made, and the procedure was adopted. We used a general surgical high-frequency electric knife with both electrotomy and electrocoagulation functions. Through a connecting cable coated with insulating material and an electrocoagulation rod, we conducted high-frequency current to the head of the electrocoagulation rod. Unipolar electrocoagulation mode coagulated and ablated target blood vessels. Using continuous mode, 30 W, energy density of about 100 J/cm (energy density is power of electrocoagulation multiplied by action time), reflected in actual operation as burning for about 3 s per retreat 1 cm, intraoperative ultrasound detected intravascular gasification as ablation target. The surgery cost about 5000CNY (about 728USD). Because the electrocoagulation rod can be reused by disinfection (like a vascular clamp), compared with the conventional RFA/EVLA cost of about 17000CNY (about 2475USD), it saves the cost of disposable consumables, about 12000CNY (about 1747USD).

## Surgical procedure, intraoperative findings, and treatment

**Figure 2:**
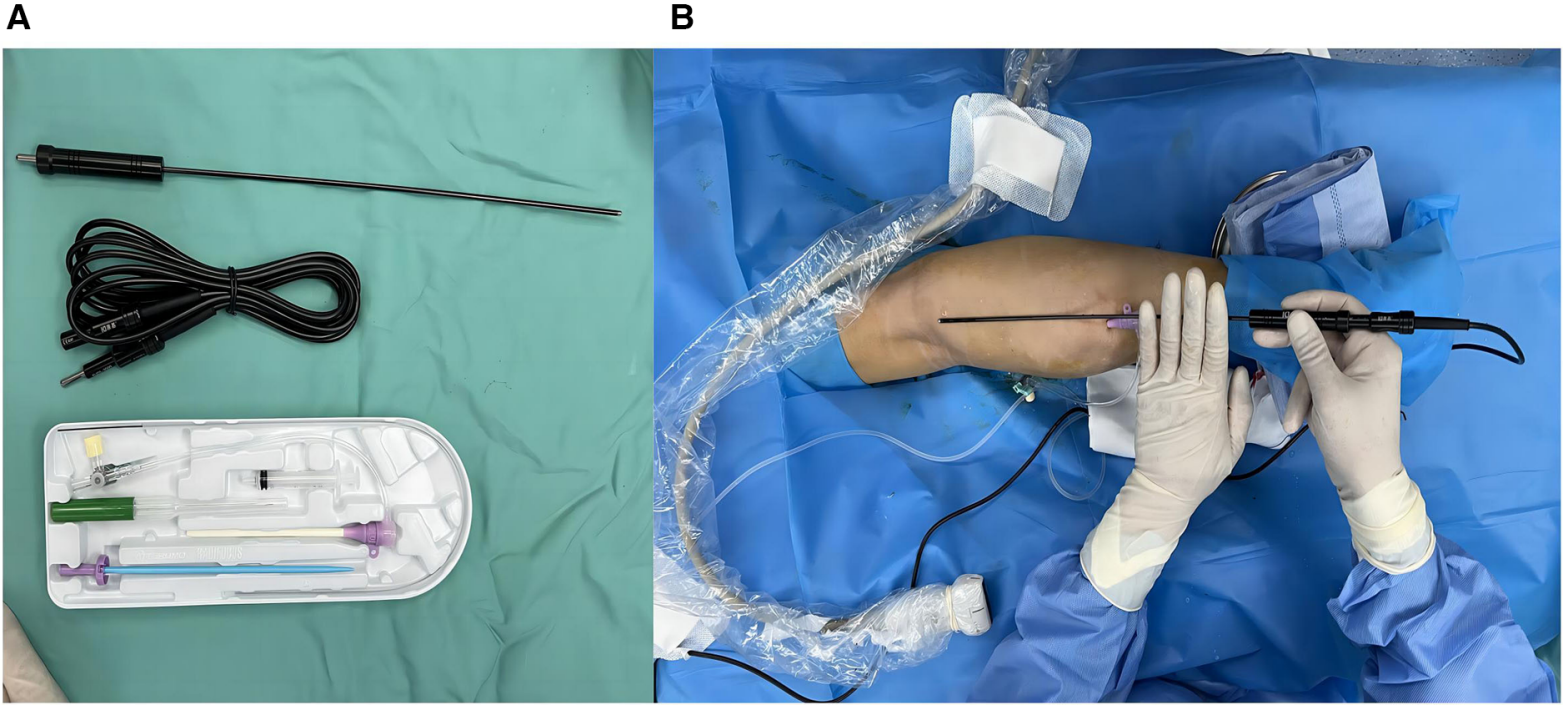
(**A**) Electrocoagulation rod and vascular sheath. (**B**) Insert the vascular sheath and roughly measure the length of insertion to the optopital junction *in vitro.*

**Figure 3:**
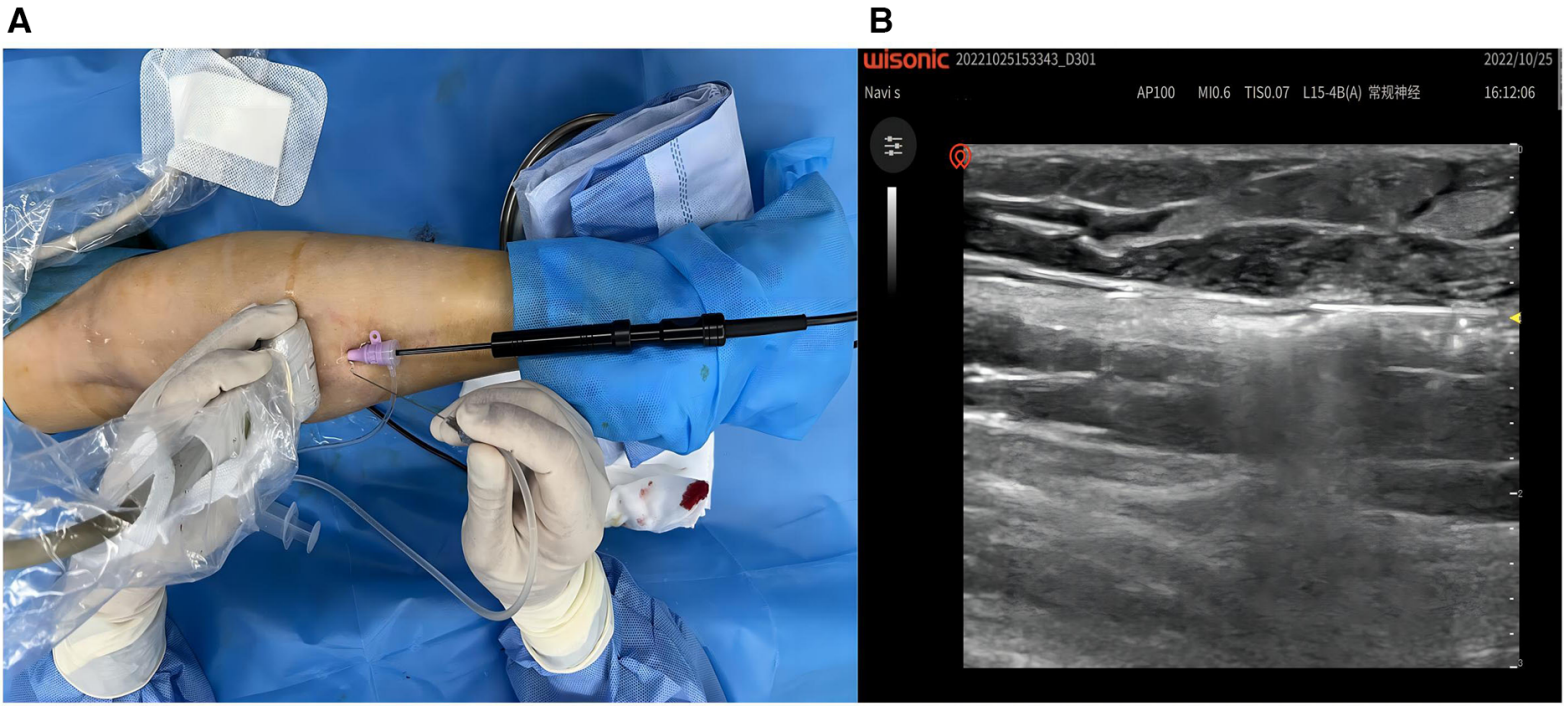
(**A**) Ultrasound-guided tumescent anesthesia. (**B**) Ablation under ultrasound monitoring.

**Figure 4:**
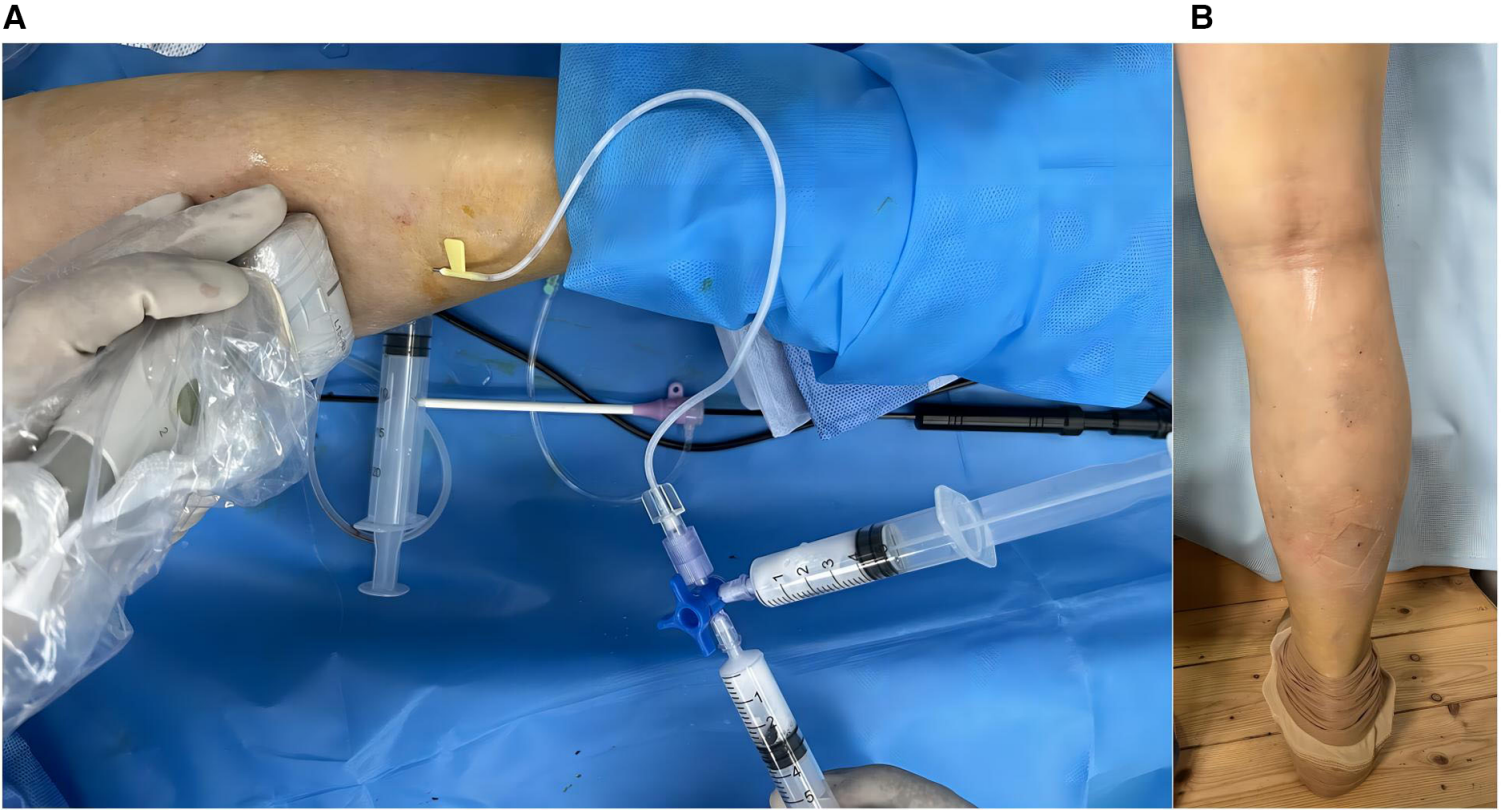
(**A**) Using foam sclerotherapy to treat varicose vein mass (**B**) Appearance 24 h after the operation.

The patient was placed in the prone position, and the surgical area was routinely disinfected and draped. After successful local anaesthesia, a 10F vascular sheath was placed in the right small saphenous vein distal to the heart with an 18G trocar cannula (lower middle 1/3 of the calf) under ultrasound guidance. An EC rod (3 mm diameter for laparoscopy) was inserted from the end of the sheath toward the heart, with a single-electrode electrocoagulation device attached to the end, and the head of the EC rod was positioned in the proximal small saphenous vein, with the beginning of the fascial compartment about 4 cm from the saphenopopliteal junction (Figure 2A, B). Under ultrasound guidance, 500 ml tumescent anaesthetic solution (470 ml 0.9% normal NaCl solution, 25 ml 1% lidocaine, 0.5 ml 0.1% epinephrine, and 5 ml 8.4% NaHCO_3_ + 20 ml 0.2% lidocaine) was injected along the saphenous fascia chamber (Figure 3A). Single-electrode EC was started at a power of 35 W with retraction of the EC rod and vascular sheath, until the small saphenous trunk above the puncture point was ablated by vaporisation (Figure 3B). Superficial varicose veins were treated with ultrasound-guided foam sclerotherapy. At the sites of severe varicose veins, vessels were punctured with a 7.5-gauge butterfly needle and foam sclerosing agent (1:4 1% polidocanol injection mixed with air) was injected locally until all varicose vessels were filled with foam (Figure 4A). At the end of the operation, the instruments and reagents were all accounted for, and the right lower extremity was wrapped with a compression bandage. Vital signs were stable during the operation. The patient recovered well and was discharged the day after surgery (Figure 4B). Oral citrus bioflavonoid tablets and level 2 compression stockings were administered for three weeks. The varicose veins disappeared, and the saphenous vein was closed at 4 cm below the saphenopopliteal junction with a VCSS score of 0 at the 3-month postoperative follow-up. There were no adverse or unanticipated events after surgery. The patients expressed their gratitude to the doctors for considering their economic situation and were satisfied with the results after the operation.

## Discussion

In current treatment protocols, endovenous thermal ablation is preferred for patients with saphenous varicose veins in the lower extremities who meet the indications for surgical treatment (3). The use of EC for thermal ablation is the better individualised treatment when economic and medical factors are considered. The high-frequency surgical electric knife, the device used to generate electricity for EC, is currently widely used in clinical practice and low in cost. Specialised devices for EC of the saphenous trunk have been developed to be used in conjunction with the electroknife (5, 7), but these devices are expensive and not available to all patients. Their interest in the exploration of electrocoagulation treatment under the premise of ensuring efficacy is worthy of recognition, because compared with the two most mainstream thermal ablation procedures, this can significantly reduce the financial burden of patients. However, no unified standard commercial EC product for ablation of the saphenous trunk is currently available on the market, and customised medical devices do not comply with legal requirements, which limits the widespread use of this type of EC procedure and hinders its development. EC rods, which are often used in laparoscopy, can solve this problem without the need for additional customised medical devices while fulfilling price and availability requirements.

EC rods are usually available in diameters of 3 mm, 5 mm, and 10 mm and can be used to stop bleeding in wounds of different diameters. The principle of EC is that when the EC head comes into contact with tissue, the change in electrical resistance generates heat energy, which then destroys the tissue and stops bleeding. Therefore, we developed the novel idea of using currently available 3-mm and 5-mm EC rods for endovenous electrothermal ablation via puncture sheaths of corresponding diameters, thus optimising therapeutic efficacy. In the present case, the patient opted not to pay for the catheter consumables required for laser or radiofrequency ablation, and after comprehensive consideration, we used a common laparoscopic EC technique for endovenous ablation, achieving the desired clinical results. EC ablation, using radiofrequency and laser ablation techniques, is contraindicated for treating certain cases of saphenous varicose veins that require surgical treatment. For example, a varicose vein with a severely dilated diameter and a thick wall is very susceptible to incomplete vessel closure after EC, and the risk of recurrence is high. The basic principles of vein stripping surgery must be strictly followed to achieve treatment efficacy, and it may be insufficient to pursue the most minimally invasive procedure alone. On the other hand, the reliability and stability of this innovative method must be further studied and observed objectively.

## Conclusion

The use of a laparoscopic EC rod as the conductive electrode of the thermal ablation electric knife in the present case may provide a reference for future improvements and developments in EC for varicose veins.

## Data Availability

The original contributions presented in the study are included in the article, further inquiries can be directed to the corresponding author.

## References

[1] Gloviczki P Comerota AJ Dalsing MC Eklof BG Gillespie DL Gloviczki ML The care of patients with varicose veins and associated chronic venous diseases: clinical practice guidelines of the society for vascular surgery and the American venous forum. J Vasc Surg. (2011) 53(5 Suppl):2S–48S. 10.1016/j.jvs.2011.01.079 21536172

[2] Gloviczki P Gloviczki ML. Guidelines for the management of varicose veins. Phlebology. (2012) 27(Suppl 1):2–9. 10.1258/phleb.2012.012s28 22312060

[3] De Maeseneer MG Kakkos SK Aherne T Baekgaard N Black S Blomgren L Editor's choice—European society for vascular surgery (ESVS) 2022 clinical practice guidelines on the management of chronic venous disease of the lower limbs. Eur J Vasc Endovasc Surg. (2022) 63(2):184–267. 10.1016/j.ejvs.2021.12.024 35027279

[4] Yoon WJ Dresher M Crisostomo PR Halandras PM Bechara CF Aulivola B. Delineating the durability outcome differences after saphenous ablation with laser versus radiofrequency. J Vasc Surg Venous Lymphat Disord. (2019) 7(4):486–92. 10.1016/j.jvsv.2018.11.013 31203857

[5] Beteli CB Rossi FH de Almeida BL Izukawa NM Onofre Rossi CB Gabriel SA Prospective, double-blind, randomized controlled trial comparing electrocoagulation and radiofrequency in the treatment of patients with great saphenous vein insufficiency and lower limb varicose veins. J Vasc Surg Venous Lymphat Disord. (2018) 6(2):212–9. 10.1016/j.jvsv.2017.09.010 29229466

[6] Rossi FH Izukawa NM Silva DG Chen J Prakasan AK Zamorano MM Effects of electrocautery to provoke endovascular thermal injury. Acta Cir Bras. (2011) 26(5):329–32. 10.1590/S0102-86502011000500001 21952653

[7] Rossi FH Beteli CB Zamorano MB Metzger PB Onofre Rossi CB Izukawa NM Experimental determination of the best time and duration for endovenous great saphenous vein electrocoagulation. J Vasc Surg Venous Lymphat Disord. (2014) 2(3):315–9. 10.1016/j.jvsv.2013.11.001 26993391

[8] Danming W Qi D Wei Y Shengjia Y Chengxun L. Clinical analysis of electrocoagulation for 27 cases of varicose veins of the lower extremities. Chin J Pract Surg. (2002) 22(2):99–100. 10.3321/j.issn:1005-2208.2002.02.015

